# Magnetic Resonance-Based Determination of Local Tissue Infection Involvement in Patients with Periprosthetic Joint Infection Following Total Hip Arthroplasty

**DOI:** 10.3390/jcm15020480

**Published:** 2026-01-07

**Authors:** Farouk Khury, Mallory Ehlers, Mark Kurapatti, Anzar Sarfraz, Vinay K. Aggarwal, Ran Schwarzkopf

**Affiliations:** 1 Department of Orthopedic Surgery, NYU Langone Health, New York, NY 10003, USA; 2 Division of Orthopedic Surgery, Rambam Health Care Campus, The Ruth and Bruce Rappaport Faculty of Medicine, Haifa 3109601, Israel

**Keywords:** total hip arthroplasty (THA), revision, peri-prosthetic joint infection (PJI), extraarticular extension, magnetic resonance imaging (MRI)

## Abstract

**Background:** Surgical treatment of periprosthetic joint infection (PJI) after total hip arthroplasty (THA) remains challenging, with thorough debridement seen as critical for success. While revision THA is well documented as the standard treatment for PJI, data on infection spread beyond the periprosthetic joint into surrounding soft tissue remain limited—this is the focus of our study. **Methods:** We retrospectively reviewed 558 patients who underwent a revision THA due to PJI at a single academic institution from January 2011 to December 2023. Out of 558 patients, 46 (8.2%) underwent a Magnetic Resonance Imaging (MRI) scan of their hip joint prior to their revision THA due to suspicion of a PJI. MRI reports were manually chart-reviewed to evaluate patients for evidence of infection spread beyond the constraints of the periprosthetic joint space. **Results:** Out of 46 patients with hip MRI prior to rTHA, 45 (97.8%) had pathological findings, and 34 (73.9%) had reports suggestive of periprosthetic joint fluid collection. The infected joint effusion extended in 30 cases (65.2%) from the capsule into the surrounding soft tissue, including the greater trochanteric region (17.4%), iliopsoas area (15.2%), anterolateral musculature (13.0%), surrounding soft tissue (10.8%), gluteal (8.7%) and obturator muscles (8.7%), and iliotibial band (4.3%). Capsule thickening was observed in 23.9% of the cases. **Conclusions:** Our study found that the vast majority (97.8%) of the hip MRIs had pathological findings, with periprosthetic joint fluid collection being a predominant feature in 73.9% of the cases. The extraarticular extension of these fluid collections was observed in over two-thirds (30/34) of affected patients, suggesting that PJI is often not confined to the capsule. MRI studies can help surgeons obtain prior knowledge of these cases and develop a more comprehensive surgical approach for infection debridement, potentially helping improve surgical treatment outcomes after PJI.

## 1. Introduction

Periprosthetic joint infection (PJI) is one of the most devastating complications following total hip arthroplasty (THA), with significant implications for patient morbidity, healthcare costs, and long-term outcomes. Despite significant advancements in joint arthroplasty, the incidence of PJI after THA is reported to be 0.4–1.4% over the past few decades, with risk persisting for the lifetime of the prosthesis and a substantial proportion of cases manifesting beyond the early postoperative period [1,2,3,4]. Risk factors for PJI include male sex, diabetes mellitus, and discharge to convalescent care, among others [2]. The increasing volume of hip arthroplasties performed worldwide has led to a parallel rise in PJI cases, underscoring the need for accurate and timely diagnosis.

Diagnosis of PJI is challenging due to the overlap of clinical symptoms with aseptic failure and the lack of universally accepted diagnostic criteria. The Musculoskeletal Infection Society (MSIS) defines PJI using major and minor criteria: the presence of a sinus tract or two positive cultures constitutes a major criterion and is diagnostic of infection, while elevated serum and synovial biomarkers are considered minor criteria [5]. However, as Hohmann et al. highlight, there remains considerable heterogeneity in PJI definitions, particularly regarding the selection and weighting of serum and synovial markers, which are often lacking methodological transparency [6].

Although microorganisms may disseminate beyond the joint via contiguous extension or hematogenous spread, current diagnostic criteria for PJI largely overlook imaging-based assessment of extraarticular involvement. Yet, the extent of soft tissue infection beyond the periprosthetic space may significantly influence surgical planning, antimicrobial strategies, and overall patient prognosis. Conventional radiography and computed tomography (CT) have limited sensitivity and specificity for PJI, often failing to distinguish infection from aseptic loosening or other causes of prosthetic failure. Nuclear medicine techniques such as leukocyte scintigraphy and fludeoxyglucose-18 (FDG) positron emission tomography (PET)-CT can identify infection and additional foci but are not routinely used due to cost and accessibility [7,8]. In contrast, emerging evidence suggests magnetic resonance imaging (MRI) may be a valuable modality to detect soft tissue manifestations of PJI extending beyond the joint capsule [9,10,11,12,13]. Specific magnetic-resonance features such as periosteal reaction, capsule edema, and intramuscular edema showed high sensitivity and specificity in the detection of PJI [9].

The American College of Radiology recommends MRI as an appropriate imaging modality in symptomatic hip arthroplasty patients when infection is suspected but not excluded, particularly for assessing extraarticular involvement [11]. MRI can detect features such as inflammatory synovitis, soft tissue edema, fluid collections, bone marrow edema, periosteal reaction, and lymphadenopathy, which are indicative of PJI. Despite these abilities, the medical literature notes that the quality of evidence regarding MRI for PJI diagnosis remains variable, with a need for consensus on standardized imaging features and larger studies to validate diagnostic criteria [14]. Nevertheless, MRI is increasingly recognized as a noninvasive, high-resolution modality that can provide critical information on extraarticular involvement in PJI after THA, informing both diagnosis and management.

Therefore, the purpose of this retrospective, hypothesis-generating study is to conduct a review of a consecutive series of patients who underwent MRI prior to revision THA for PJI to further evaluate the utility of MRI in characterizing the extraarticular extent of periprosthetic joint infections. We hypothesize that MRI can reveal significant extraarticular soft tissue involvement in a substantial proportion of patients, which has the potential to influence surgical planning and clinical outcomes. Ultimately, our goal is to support the integration of MRI as a diagnostic and preoperative tool, thereby enabling further optimization of clinical management of PJI.

## 2. Methods

### 2.1. Study Design

**Figure 1:**
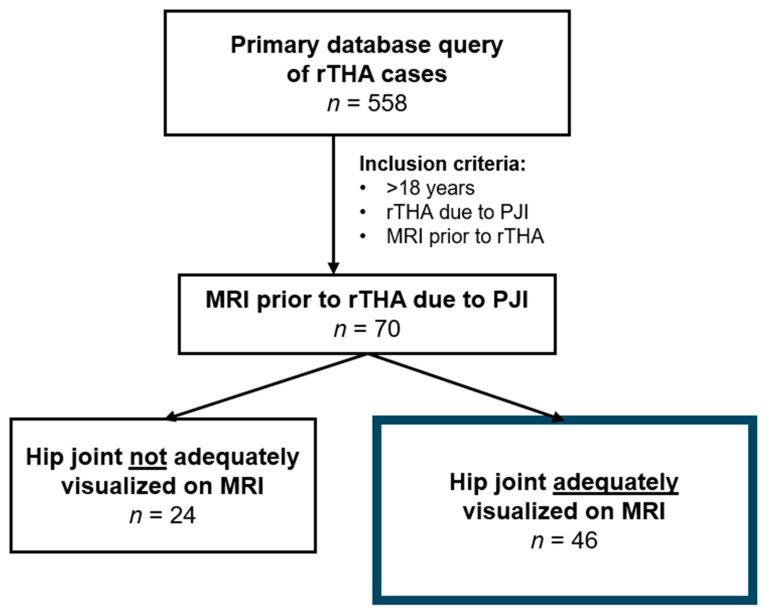
Flow chart demonstrating patient selection. rTHA, Revision total hip arthroplasty; PJI, Periprosthetic joint infection; *n*, Number.

A retrospective review, with an Institutional Review Board-approved research protocol, was conducted of patients who underwent reoperation or revision after THA due to PJI at a single academic institution from January 2011 to December 2023. Primary query yielded 558 eligible patients. Patients were included in the study if they were older than 18 years of age and had undergone THA that required subsequent debridement and irrigation with exchange of modular implant parts (DAIR) or two-stage revision arthroplasty after MRI of the hip joint for a suspected PJI. Patients whose MRI scans did not adequately visualize the hip joint were excluded from the study. Of the 558 patients, 70 (12.5%) had undergone MRI prior to the revision THA. Of the 70 patients, 24 were excluded due to MR-imaging that did not include the hip joint region. The final study cohort consisted of 46 patients (Figure 1).

### 2.2. Imaging Protocol

**Figure 2:**
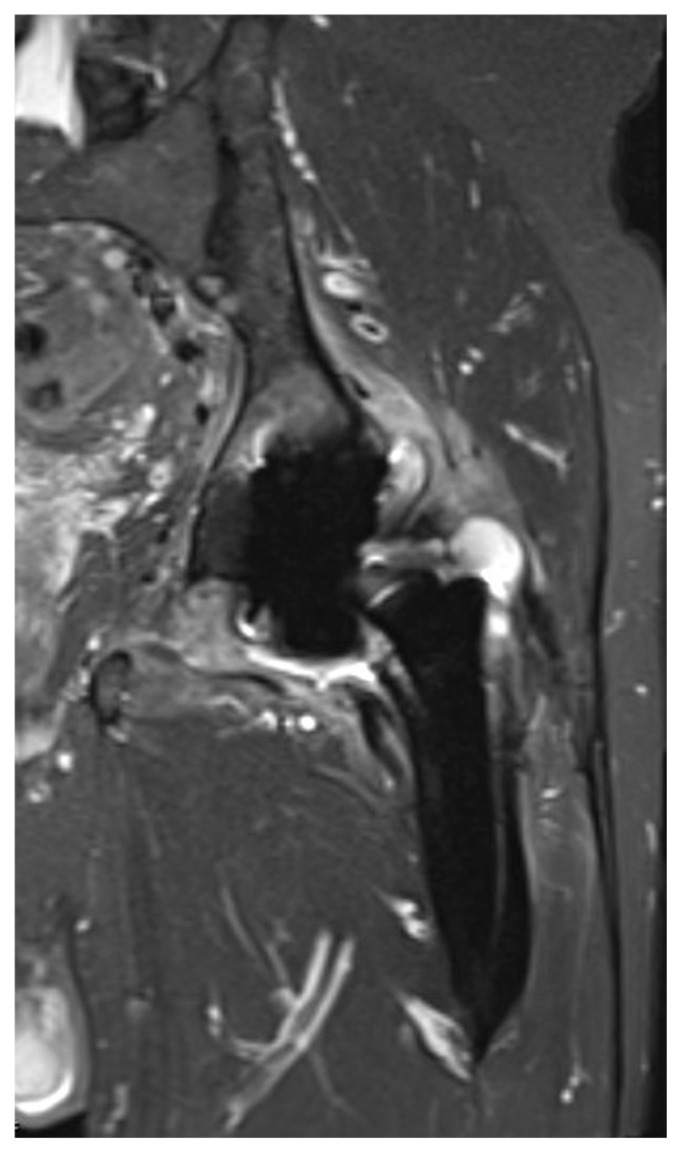
Coronal MRI of the left hip demonstrating signs suggestive of PJI, including a large, decompressing joint effusion, significant capsular enhancement, and extensive periarticular muscle edema. The presence of bone marrow edema at the metal-bone interface (Gruen zones) further suggests septic loosening of the components.

MRI was performed on either a 1.5-T or 3-T system using dedicated protocols designed to minimize metal artifacts caused by the implant components (Figure 2). All MR images were acquired using Metal Artifact Reduction Sequences (MARS) [15], such as Multi-Acquisition Variable-Resonance Image Combination (MAVRIC) or similar T2-weighted turbo spin-echo sequences, to optimize the depiction of periprosthetic soft tissues and bone. All patients in the study received intravenous contrast (gadolinium-based) as part of the standard imaging protocol to help differentiate between phlegmon and abscess, and to better delineate sinus tracts. Patients with a metal-on-metal bearing surface were not included in this study.

### 2.3. Data Collection

Our institution’s EMR (Epic Caboodle. Version 15; Verona, WI, USA) was utilized to collect patients’ demographics (e.g., gender, age, body-mass index (BMI), past medical and surgical histories, operative reports for DAIR and two-stage revision arthroplasty, and complete MRI reports. We also collected data on the indication for rTHA, the type of treatment (DAIR or two-stage revision), the surgical approach used (anterior-, lateral-, or posterior-based approach), whether a single or dual incision was used, failure of treatment, subsequent treatment following failure, and the microbiological profile based on final culture results.

MRI sequences were initially interpreted and reported by a dedicated team of specialized musculoskeletal radiologists with experience in post-arthroplasty imaging following a standardized institutional imaging protocol. Pathological findings suggestive of infection were identified based on established criteria, including the presence of lamellated hyperintense synovitis, extracapsular fluid collections, periosteal reaction, and intramuscular edema [9,10,16]. The resulting MRI reports were systematically reviewed by the orthopedic surgeon to evaluate pathological findings. The variables extracted from the reports included the specific location and extent of infection spread beyond the constraints of the periprosthetic joint space, joint effusion (presence, amount, and location of periprosthetic joint fluid collections), and pathological findings such as osteomyelitis, sinus tract, edematous changes (specifically in the greater trochanter region, iliopsoas, sacroiliac joint, and adjacent soft tissue/musculature), and capsule thickening.

### 2.4. Statistical Analysis

Continuous variables were reported as means with standard deviations, and categorical variables were expressed as frequencies and percentages. Due to the descriptive nature of this study and the sample size, formal association testing between specific MRI findings and clinical outcomes was not performed.

## 3. Results

### 3.1. Patient Demographics

The 46 patients investigated were aged 64.3 ± 9.9 years and had a body mass index of 31.7 ± 6.7. Of the entire cohort, 45.7% were female and 54.3% were male. The majority of the patients were White (73.9%), non-smokers (50.0%), had an ASA score II (52.2%), underwent the revision surgery under general anesthesia (67.4%), and had a mean Charlson Comorbidity Index (CCI) of 3.5 ± 2.0. Length of stay was 7.3 ± 6.6 days and patients’ average follow-up was 25.3 ± 25.4 months. Patient demographic parameters are presented in Table 1.

### 3.2. Operative Characteristics

Table 2 demonstrates the operative characteristics, microbiological profiles and surgical outcomes of the study cohort. The majority of the rTHAs were due to acute PJI (*n* = 23, 50%), followed by chronic PJI (*n* = 20, 43.5%), and unknown etiology (*n* = 3, 6.5%). The intraoperative bacterial cultures demonstrated growth of methicillin-sensitive *Staphylococcus aureus* (MSSA) (*n* = 22, 47.8%), followed by culture-negative results (*n* = 13, 28.3%), multiple organisms (*n* = 6, 13.0%), *Escherichia coli* (*n* = 3, 6.5%), methicillin-resistant *Staphylococcus aureus* (MRSA) (*n* = 3, 6.5%), *Morganella morganii* (*n* = 1, 2.2%), *Abiotrophia defectiva* (*n* = 1, 2.2%), and Candida species (*n* = 1, 2.2%). The rTHAs consisted of DAIR (*n* = 24, 52.2%) and two-stage revision arthroplasty (*n* = 22, 47.8%). The rTHAs were performed mostly using a posterior-based approach (*n* = 41, 89.1%), followed by an anterior-based approach (*n* = 3, 6.5%), and a lateral-based approach (*n* = 2, 4.3%). The majority of the surgeries were performed using a single incision (*n* = 37, 80.4%) rather than dual incisions (*n* = 9, 19.6%). Of the 46 rTHAs, 13 (28.3%) failed and required either two-stage revision arthroplasty (*n* = 9, 69.2%), one-stage revision arthroplasty (*n* = 2, 15.4%) or DAIR (*n* = 2, 15.4%). The surgical approaches utilized in the consequent rTHA were posterior-based (*n* = 10, 76.9%), lateral-based (*n* = 2, 15.4%), and anterior-based (*n* = 1, 7.7%).

### 3.3. Imaging Findings

Out of 46 patients who had pre-rTHA MRI adequately demonstrating the hip joint, 97.8% exhibited some sort of pathological finding, mostly in the greater trochanter region (23.9%). 73.9% of the entire cohort had MRI reports describing periprosthetic joint fluid collections, mainly in moderate (47.8%), followed by large (17.4%), and small (8.7%) amounts. Extraarticular joint fluid extensions into surrounding anatomical locations were seen in 65.2% of the cases. The extensions were to the greater trochanter region (17.4%), iliopsoas muscle (15.2%), anterolateral musculature (13.0%), surrounding soft tissue (10.8%), gluteal (8.7%) and obturator muscles (8.7%), and iliotibial band (4.3%). Patients who did not have joint effusion (26.1%) exhibited other pathological findings in their MRI, such as sinus tract (10.8%), osteomyelitis (2.2%), and edematous changes in the greater trochanter region (6.5%), soft tissue and musculature near the hip joint (4.3%), iliopsoas muscle (2.2%), and sacroiliac joint (2.2%). Capsule thickening and signs of synovitis were seen in 23.9% of the cases. Table 3 demonstrates the MRI-based characterization and anatomical distribution of the PJI.

## 4. Discussion

PJI following THA remains a dreadful complication, necessitating meticulous surgical management to achieve optimal outcomes. While extensive literature highlights the significance of thorough debridement and revision surgery in treating PJI, the potential for infection to extend beyond the periprosthetic joint space into adjacent soft tissue has been relatively underexplored. Our descriptive results demonstrate that extraarticular extension of joint fluid was observed in over two-thirds of the affected patients, highlighting the potential role of preoperative MRI in identifying such infection extensions, which may optimize surgical planning and improve outcomes.

While previous studies [9,10,16] primarily focused on MRI as a diagnostic tool for detecting PJI, our study emphasizes its utility in defining the extent of infection in already diagnosed cases. Importantly, MRI provides superior visualization of soft tissues and can detect infection-related fluid collections, abscesses, and muscle involvement, which may not be fully appreciated through standard imaging modalities such as plain radiographs or computer-tomographic scans [8,9]. This visualization is critical for preoperative planning, as the extent of infection beyond the joint capsule may necessitate more extensive surgical debridement or influence the choice of antimicrobial therapy. Furthermore, MRI has the advantage of not using ionizing radiation or contrast agents [17,18]. However, while MRI can be performed without contrast, all patients in this cohort received intravenous gadolinium-based contrast as part of our institutional protocol to better delineate sinus tracts and differentiate phlegmon from abscess. The American College of Radiology notes that intravenous contrast may aid in differentiating phlegmon from abscess and delineating sinus tracts, but non-contrast MRI is often sufficient for diagnosis [11].

The ability to preoperatively assess the extent of the infection and involvement of the surrounding soft tissue, particularly when applying MARS [17,19], can aid surgeons in planning a more comprehensive debridement strategy, potentially improving surgical outcomes and reducing recurrence rates. Identification of extraarticular extension may alter the surgical approach, prompt more aggressive debridement, or necessitate multidisciplinary management, including infectious disease consultation for tailored antimicrobial therapy. MRI can also aid in the differentiation of PJI from other causes of prosthetic failure, such as aseptic loosening or particle disease, thereby preventing unnecessary revision surgery and optimizing patient outcomes. While our study focused on a cohort with confirmed PJI, the observed MRI features such as sinus tracts and specific edema patterns are noted in the literature [9,10,16] to aid in this differentiation.

The findings from our infected cohort indicate that PJIs frequently extend into crucial musculotendinous structures, potentially impacting joint stability, postoperative recovery, risk of reinfection, and complexity of surgical management. Involvement of the greater trochanteric region, iliopsoas, anterolateral musculature, gluteal and obturator muscles, and iliotibial band can compromise abductor and flexor function, leading to this instability and impaired postoperative recovery. Therefore, the implications of infection extension into periarticular soft tissues are clinically significant, as the management of soft tissue is a critical step in the treatment of PJI [20,21]. Infection in these areas may necessitate more extensive debridement and can increase the risk of persistent infection and recurrence, as soft tissue reservoirs are difficult to eradicate and may harbor biofilm-forming organisms [22]. Accordingly, studies have suggested that soft tissue involvement in PJI is associated with a higher risk of persistent infection, surgical complications, and the need for more extensive debridement [20,21]. Therefore, infected anatomical locations ought to be adequately identified prior to revision surgery.

As previously discussed, MRI is highly sensitive for detecting edema, abscesses, and fluid collections in these soft tissue compartments, enabling precise preoperative mapping of infection spread. MRI findings such as intramuscular edema and capsule thickening are strong discriminators of PJI and help identify which anatomical regions require targeted debridement. In our study, MRI demonstrated that 97.8% of the entire cohort had some sort of pathological finding visible on MR imaging. Furthermore, infected joint fluid effusion into extraarticular locations was demonstrated in 73.9% of the cases investigated in our study. This finding highlights the fact that a meticulous review of the MRI scan can help the surgeon in preoperative planning.

The infected cohort investigated in this study exhibited extracapsular infection extension into the greater trochanteric region, iliopsoas, anterolateral musculature, surrounding soft tissue, gluteal and obturator muscles, and iliotibial band. Although these regions can be accessible through a number of approaches, certain anatomical areas present unique surgical challenges when involved in infection. Our finding that 15.2% of cases exhibited involvement of the iliopsoas region is particularly significant, as this area is difficult to access due to its deep location and proximity to critical neurovascular structures [23]. Other than being the largest synovial bursa in the body, the iliopsoas bursa is an important anatomical structure that has been shown to communicate with the native hip joint and pseudo-capsule [24,25]. While the iliopsoas region can be accessed through multiple surgical routes—including extraperitoneal, transperitoneal, or retroperitoneal approaches—the selection of the optimal corridor depends heavily on the precise extent of the infection and patient anatomy [26,27]. Preoperative identification of such involvement via MRI is therefore essential to guide the surgeon in selecting the most appropriate surgical approach for thorough debridement while minimizing risks to adjacent neurovascular structures [23].

Debridement of an infection involving the obturator muscles, in particular the obturator internus, is another challenging surgical procedure due to the proximity of critical neurovascular structures and the anatomical variability of the obturator to iliac (epigastric) anastomosis. The ilioinguinal and Pfannenstiel approaches, while commonly used, require careful dissection to avoid injury to the obturator nerve and vessels [28,29,30,31,32]. Furthermore, infection extension as well as surgical debridement of the gluteal musculature, greater trochanter and iliotibial band may compromise hip abductor function, which is critical for postoperative mobility and gait stability [33,34,35]. Anatomical studies have shown that the obturator internus lies deep within the pelvis, and its involvement in infection can necessitate extended exposure and meticulous technique to achieve adequate debridement while minimizing complications. The risk of neurovascular injury is heightened by the presence of infection-related inflammation and tissue distortion, which can obscure normal anatomical landmarks and increase surgical difficulty [36]. Additionally, the gluteal muscles, particularly the gluteus medius and minimus, are primary stabilizers of the hip, and their involvement in infection may result in muscle necrosis, weakness, or detachment. Surgical management in these cases often requires not only removal of infected tissue but also consideration of reconstructive techniques to restore abductor function and prevent postoperative instability [37,38]. All these factors highlight the need for meticulous preoperative imaging to identify the anatomical spread of the infection and help plan an extended surgical approach to address the infection.

Given the high prevalence of soft tissue extension identified in the study cohort, with 65.2% of patients undergoing MRI showing extraarticular involvement and over two-thirds demonstrating joint fluid collections, it is imperative for surgeons to incorporate advanced imaging into the preoperative assessment of complex PJI cases. MRI enables precise localization of infection, identification of abscesses, and delineation of anatomical spread, which informs the surgical approach and extent of debridement required. Incorporating these imaging findings into surgical planning allows for a more aggressive and targeted intervention, including extended soft tissue debridement, targeted antibiotic therapy, and potential modification to implant selection and fixation strategies. Therefore, the addition of MRI to preoperative assessment has the potential to improve infection eradication rates and functional outcomes.

Management of soft tissue infection is a cornerstone of successful PJI treatment, as adequate debridement and preservation of viable tissue are essential for restoring blood supply, which is crucial for local reconstruction and healing, and reducing the risk of persistent infection [21,39]. Collaboration among orthopedic surgeons, infectious disease specialists, and musculoskeletal radiologists is necessary to optimize treatment strategies, interpret imaging findings, and tailor antibiotic therapy to the specific anatomical and microbiological context of each case [40,41]. This multidisciplinary approach is supported by consensus in the medical literature as a means to improve patient outcomes and reduce complications associated with complex PJI cases.

This study is not without limitations. First, this study is limited by its retrospective nature, which may introduce inherent biases such as selection bias or incomplete data. Second, the relatively small sample size investigated may limit the generalizability of our findings. Additionally, the small sample size precluded the use of multivariate models to adjust for confounders such as microorganism type, culture results, or duration of infection. Third, MRI findings were based on radiological reports rather than standardized quantitative measures of infection spread, which may introduce variability in interpretation. Systematic reviews have highlighted variability in the sensitivity and specificity of MRI features, such as capsule edema, periosteal reaction, and intramuscular edema, with accuracy ranging from 63.9% to 94.4% and interobserver agreement varying widely [14]. There is currently no consensus on the definition or weighting of specific MRI findings for PJI diagnosis, and large-scale, high-quality studies are needed to establish robust criteria [42,43]. Additionally, optimized MRI protocols are not universally available, and image quality can vary depending on scanner type, field strength, and operator expertise [44]. Fourth, the decision to perform MRI was based on clinical judgement and suspicion of soft tissue involvement, meaning that MRI may not have been applied uniformly across all patients, which introduces a significant selection bias and may influence the generalizability of our findings. Our findings may be more applicable to complex or advanced PJI cases—such as those with persistent symptoms, unclear sinus tracts, or suspected deep soft-tissue involvement—rather than routine PJI presentations. Fifth, this study is observational in nature and does not provide a direct comparison between patients who underwent MRI and those who did not. Overall, observational studies may overestimate associations and cannot establish causality, as highlighted in orthopedic research methodology literature [45,46]. The generalizability of the findings may be limited by the single-center setting, patient selection criteria, and retrospective data collection. It is possible that patients who underwent MRI had more complex or advanced infections, leading to more extensive surgical interventions, which introduces inherent limitations such as selection bias, confounding, and lack of randomization. As such, the findings may be more applicable to cases where MRI is already being used in clinical practice rather than to all PJI cases. Sixth, this study exclusively focused on MRI as an imaging modality and did not consider other imaging techniques, such as plain radiographs, CT or ultrasound. Each of these modalities provides distinct advantages and limitations in the evaluation of PJI. For instance, plain radiographs are useful in detecting structural changes and implant-rated issues, while CT scans provide better visualization of bone and joint anatomy. Ultrasound, on the other hand, is a cost-effective, fast modality that can be valuable for detecting superficial fluid collections and soft tissue abnormalities [8]. Future studies ought to compare multiple imaging techniques in preoperative evaluation of PJI extension to determine the accuracy and utility of these additional modalities. Seventh, clinical management decisions should not rely solely on MRI findings. The diagnosis and treatment of PJI require integration of clinical, laboratory, microbiologic, and imaging data, as recommended by the Infectious Diseases Society of America [47] and the American College of Radiology [11]. As emphasized by the Infectious Diseases Society of America, microbiologic diagnosis requires synovial fluid and tissue cultures, ideally obtained intraoperatively and processed using standardized protocols. Imaging findings alone cannot distinguish between different organisms or confirm polymicrobial infection, and false positives may occur due to non-infectious causes of soft tissue edema or fluid collections. Therefore, multidisciplinary collaboration remains essential to optimize patient outcomes. Lastly, the impact of MRI findings on surgical outcomes and long-term patient prognosis remains uncertain. While our study identifies the extent of soft tissue involvement, further research is needed to determine whether preoperative identification of infection spread via MRI directly translates into improved surgical outcomes, reduced recurrence rates, or better functional recovery. Future studies could examine whether incorporating MRI findings into surgical planning leads to a reduction in complications, faster recovery times, or improved functional outcomes in patients undergoing revision THA for PJI.

In conclusion, our descriptive study characterizes the prevalence and clinical significance of MRI-based soft tissue extension in this cohort of PJI cases. MRI identifies extracapsular infection spread to guide surgical approach selection, the extent of debridement, and the use of reconstructive techniques when musculotendinous structures are involved. Integrating these findings into decision-making may improve diagnostic accuracy and reduce the risk of recurrence, although further research is required to determine if this directly improves patient outcomes. Future prospective, multicenter studies are needed to validate the prognostic value of MRI and develop standardized reporting frameworks. Ultimately, these efforts support evidence-based algorithms to optimize surgical planning and long-term recovery in revision THA for PJI.

## Figures and Tables

**Table 1:** Patient demographics and clinical characteristics.

Parameter	Cohort (*n* = 46)
Mean age at surgery ± SD [range], (years)	64.3 ± 9.9 [33–81]
Mean BMI ± SD [range], (kg/m^2^)	31.7 ± 6.7 [17.7–44.3]
Sex, *n* (%)	
Female	21 (45.7)
Male	25 (54.3)
Race, *n* (%)	
White	34 (73.9)
Black or African American	7 (15.2)
Hispanic	2 (4.3)
Other	3 (6.5)
Smoking status, *n* (%)	
Never	23 (50.0)
Former	19 (41.3)
Current	4 (8.7)
ASA score, *n* (%)	
I	0 (0.0)
II	24 (52.2)
III	21 (45.7)
IV	1 (2.2)
Anesthesia type, *n* (%)	
General	31 (67.4)
Spinal	15 (32.6)
Mean CCI ± SD	3.5 ± 2.0
Mean length of stay ± SD, (days)	7.3 ± 6.6
Mean length of follow-up ± SD, (months)	25.3 ± 25.4

SD, Standard deviation; *n*, Number; %, Percentage; BMI, Body-mass index; ASA, American Society of Anesthesiologists; CCI, Charlson Comorbidity Index.

**Table 2:** Operative characteristics, microbiological profiles, and surgical outcomes of the study cohort.

Parameter	Cohort (*n* = 46)
Indication for rTHA, *n* (%)	
Acute PJI	23 (50.0)
Chronic PJI	20 (43.5)
Unknown	3 (6.5)
Growth of cultures, *n* (%)	
MSSA	22 (47.8)
*Escherichia coli*	3 (6.5)
*Morganella morganii*	1 (2.2)
MRSA	3 (6.5)
*Enterobacter cloacae*	2 (4.3)
*Abiotrophia defectiva*	1 (2.2)
*Candida species*	1 (2.2)
Culture-negative	13 (28.3)
Treatment, *n* (%)	
DAIR	24 (52.2)
Two-stage revision arthroplasty	22 (47.8)
Surgical approach, *n* (%)	
Anterior-based	3 (6.5)
Lateral-based	2 (4.3)
Posterior-based	41 (89.1)
Single or dual incisions, *n* (%)	
Single	37 (80.4)
Dual	9 (19.6)
Failure of treatment, *n* (%)	13 (28.3)
Treatment following failure, *n* (%)	
DAIR	2 (15.4)
One-stage revision arthroplasty	2 (15.4)
Two-stage revision arthroplasty	9 (69.2)
Surgical approach following treatment failure, *n* (%)	
Anterior based	1 (7.7)
Lateral based	2 (15.4)
Posterior based	10 (76.9)

rTHA, Revision total hip arthroplasty; DAIR, Debridement, antibiotics, and implant retention; MSSA, Methicillin-sensitive *Staphylococcus aureus*; MRSA, Methicillin-resistant *Staphylococcus aureus*; *n*, Number; %, Percentage.

**Table 3:** Magnetic resonance imaging-based characterization and anatomical distribution of the pathological findings.

Parameter	Cohort (*n* = 46)
Any pathological finding, *n* (%)	45 (97.8)
Location	
Anterolateral musculature	6 (13.0)
Greater trochanter	11 (23.9)
Iliotibial band	2 (4.3)
Gluteal muscles	4 (8.7)
Iliopsoas muscle	8 (17.4)
Obturator muscles	4 (8.7)
Sacroiliac joint	1 (2.2)
Soft tissue and musculature adjacent to hip joint	7 (15.2)
Joint effusion, *n* (%)	34 (73.9)
Amount	
Small	4 (8.7)
Moderate	22 (47.8)
Large	8 (17.4)
Location extension	30 (65.2)
Anterolateral musculature	6 (13.0)
Greater trochanter	8 (17.4)
Iliotibial band	2 (4.3)
Gluteal muscles	4 (8.7)
Iliopsoas muscle	7 (15.2)
Obturator muscles	4 (8.7)
Surrounding soft tissue and musculature	5 (10.8)
No joint effusion, *n* (%)	12 (26.1)
Pathological finding	
Sinus tract	5 (10.8)
Osteomyelitis	1 (2.2)
Edematous changes	
Greater trochanter	3 (6.5)
Iliopsoas	1 (2.2)
Sacroiliac joint	1 (2.2)
Soft tissue and musculature adjacent to hip joint	2 (4.3)
Capsule thickening, *n* (%)	11 (23.9)

*n*, Number; %, Percentage.

## Data Availability

The data presented in this study are available on request from the corresponding author. The data are not publicly available due to privacy and ethical restrictions.
